# Lung cancer survival among Florida male firefighters

**DOI:** 10.3389/fonc.2023.1155650

**Published:** 2023-08-18

**Authors:** Tulay Koru-Sengul, Paulo S. Pinheiro, Wei Zhao, Monique N. Hernandez, Diana R. Hernandez, Alessandra Maggioni, Erin N. Kobetz, Alberto J. Caban-Martinez, David J. Lee

**Affiliations:** ^1^ Department of Public Health Sciences, University of Miami, Leonard M. Miller School of Medicine, Miami, FL, United States; ^2^ Sylvester Comprehensive Cancer Center, University of Miami, Leonard M. Miller School of Medicine, Miami, FL, United States; ^3^ Florida Cancer Data System, University of Miami, Leonard M. Miller School of Medicine, Miami, FL, United States; ^4^ Department of Medicine, University of Miami, Leonard M. Miller School of Medicine, Miami, FL, United States; ^5^ Department of Physical Medicine and Rehabilitation, University of Miami, Leonard M. Miller School of Medicine, Miami, FL, United States

**Keywords:** lung cancer, cancer survival, firefighters, Florida, occupational exposure

## Abstract

**Introduction:**

Lung cancer is a leading cause of cancer incidence and death in the United States. Although most firefighters are fit and do not smoke, they are exposed to many known carcinogens during and in the aftermath of firefighting activities. Comprehensive epidemiologic investigations on lung cancer survival for both career and volunteer firefighters have not been undertaken.

**Methods:**

Data from the Florida Cancer Data System (1981–2014) were linked with firefighter certification records from the Florida State Fire Marshal’s Office to identify all patients of this occupational group; lung cancer cause-specific survival data were compared with other occupational groups using Cox regression models with occupation as the main effect. Adjusted hazard ratios (aHR) and 95% confidence intervals (95% CI) were calculated.

**Results:**

Out of 210,541 male lung cancer cases diagnosed in Florida (1981–2014), 761 were firefighters (604 career, 157 volunteer). Lung cancer death was similar between volunteer (75.2%) and career firefighters (74.0%) but lower than non-firefighters (80.0%). Survival at 5 years was higher among firefighters (29.7%; career: 30.3%; volunteer: 27.4%) than non-firefighters (23.8%). In a multivariable model, compared with non-firefighters, firefighters have significantly higher cause-specific survival (aHR = 0.84; 95% CI: 0.77–0.91; *p* < 0.001). However, there were no significant survival differences between career and volunteer firefighters (1.14; 0.93–1.39; *p* = 0.213). In a separate multivariable model with firefighters as the comparator, other broad occupational groups had significantly lower cause-specific survival [white collar: 1.11 (1.02–1.21); blue collar: 1.15 (1.05–1.25); service: 1.13 (1.03–1.25); others/unknown: 1.21 (1.12–1.32); all *p*-values < 0.02].

**Conclusion:**

Lung cancer survival is significantly higher among firefighters compared with non-firefighters, but there is no significant difference between career and volunteer firefighters. Improved survival for firefighters might be due to a healthy worker effect, lower smoking prevalence relative to other worker groups, and possibly superior treatment adherence and compliance. Many firefighters are cross-trained as EMTs/paramedics and possess a level of medical knowledge that may favorably impact treatment engagement and better navigation of complex cancer care.

## 1 Introduction

Lung cancer is the most predominant cause of cancer-specific death in the United States (US), in addition to being the second leading cause of death overall (1, 2). Lung cancer accounts for 20% of cancer deaths, making it the primary cause of death among all cancers (1). In 2023, it is anticipated that approximately 238,340 new cases of lung cancer (117,550 in men and 120,790 in women) with 127,070 deaths (67,160 in men and 59,910 in women) in the US are expected (1, 3, 4). The age-adjusted lung cancer death rate in the US was 34.8 per 100,000 in 2018 (5). Notably, the age-adjusted incidence and death rate for lung cancer was higher in men than in women (incidence: 75.2 *vs*. 52.3 and death: 46.7 *vs*. 31.9; per 100,000) (6). In the US, the 5-year relative survival rate for lung cancer is 18.6%, which is lower than any other major cancer site (7). The highest age-adjusted lung cancer death rate was reported to be 53.5 per 100,000 (8). The 5-year relative survival rate varies by lung cancer type and tumor stage (8). The rates for non-small cell lung cancer (NSCLC) are substantially higher than the rates for small cell lung cancer (SCLC) (overall 28% *vs*. 7%; localized 65% *vs*. 30%; regional 37% *vs*. 18%; distant 9% *vs*. 3%, respectively) (8). Florida ranks 22nd in the nation for lung cancer incidence with 56 per 100,000 compared with the national 57 per 100,000 (9). Moreover, 25% of all cases are detected at an early stage. Furthermore, Florida’s 5-year relative survival rate is 26.5%, which is markedly greater than the nationwide survival rate of 25%, ranking 14th in the nation (9).

The burden of lung cancer incidence and mortality is reflective of the pervasiveness of its risk factors. Approximately 80% of lung cancer cases can be attributed to cigarette smoking (10). However, individuals that do not engage in tobacco use may still develop lung cancer. Risk factors that do not include tobacco usage include secondhand smoke exposure, radon exposure, and exposure to cancer-causing agents in the workplace, among others. Occupational exposures to known carcinogens, which can be classified as preventable causes, have been identified in different areas of work, particularly in firefighting (11, 12).

Firefighters are exposed to various toxic substances by the inhalation of particulate matter and gases. They are susceptible to significant amounts of carbon monoxide, benzene, sulfur dioxide, hydrogen cyanide, acrolein, aldehydes, hydrogen chloride, nitrogen dioxide, chlorinated hydrocarbons, trichloroethylene, toluene, dichlorofluoromethane, and soot. Health surveys have shown that firefighters have an increased prevalence of respiratory symptoms and reduced lung function due to exposure to toxic substances by inhalation of particulate matter and gases. Studies have shown that firefighters have an increased risk of cancer due to occupational prolonged bronchial epithelium exposure to inhaled particles and carcinogens (13, 14). There exists sufficient evidence showing that firefighters are exposed to a range of cancer-causing toxins on the job (15–18). As a result of such evidence, in the summer of 2022, the World Health Organization’s International Agency for Research on Cancer (IARC) reclassified firefighting as a carcinogenic profession, and an IARC Group 1 designation was given (19, 20). Furthermore, firefighters are also at increased risk of dying from non-malignant respiratory diseases. While respiratory protection is widely available, the nature of their job makes proper protection difficult to ensure (21).

Despite these exposure risks, firefighters appear not to be at increased risk of lung cancer. Based on a systematic review of case–control studies, there was no increased lung cancer risk overall or by specific cell type among firefighters with and without smoking status adjustment (22). There were also no significant exposure–response relationships in terms of work duration. Other investigators have reported that some lung cancers that develop in never-smoking career firefighters should be considered potentially job-related (23).

There is no known study in the literature on the lung cancer survival experience of firefighters. Comparing the lung cancer characteristics and the survival of firefighters to non-firefighters and of different occupations will help fill this knowledge gap. The current study has two major contributions. First, we aim to assess specific patterns of lung cancer in firefighters including demographics such as smoking status at the time of cancer diagnosis, tumor characteristics such as histology, and cancer treatment. Second, we aim to evaluate cancer survival among a group of Florida male firefighters (career and volunteer), as cancer survivors of the first primary lung cancer compared with the Florida general population of non-firefighters with different occupational histories over a 33-year period (1981–2014). This study will provide the first population-based analysis of the epidemiological analysis of lung cancer survival among male firefighters in Florida.

## 2 Materials and methods

### 2.1 Study design

This study is part of a retrospective observational cohort from a broader data linkage project as part of the Firefighters Cancer Initiative (24, 25).

### 2.2 Data sources

This study uses the Florida Cancer Data System (FCDS) incidence cancer records (1981–2014) as the analytical cohort. This dataset linked data from three additional data sources: 1) firefighter certification records from the Florida State Fire Marshal’s Office (FMO) (1972–2012); 2) LexisNexis©, a national dataset of legal, government, business, and high-tech information for identification of missing linkage variables (e.g., date of birth, social security number); and 3) Florida Office of Vital Statistics and the National Death Index (1981–2019) for computation of survival time from cancer diagnosis. The design and methods of the original data linkage with the first three sources of data have been published previously (24).

### 2.3 Study population

**Figure 1:**
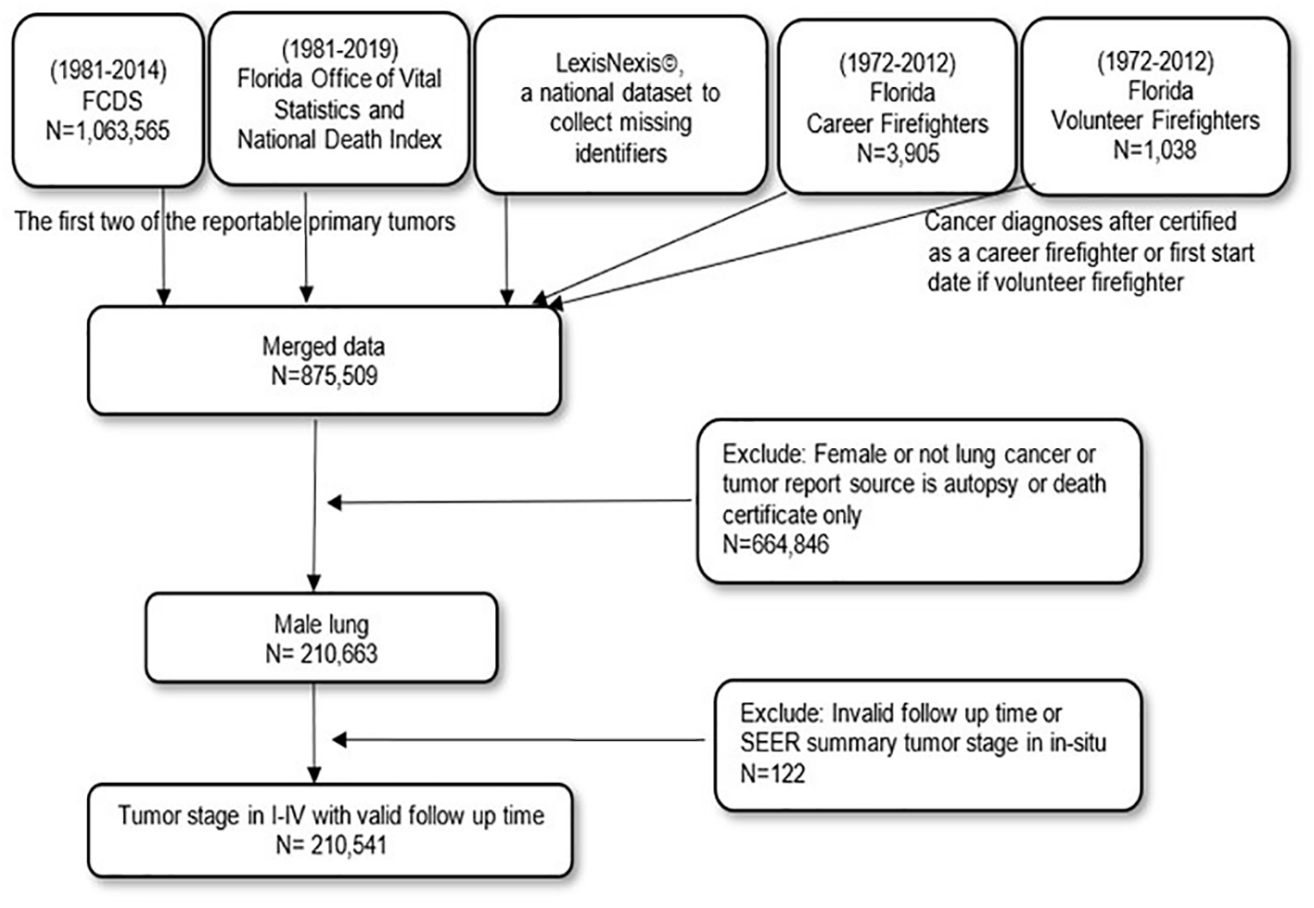
The data linkage flowchart from individual data sources to the final analytical dataset for male lung and bronchus cancer in Florida.

From the linked dataset, male patients in Florida with a diagnosis of primary or first multiple primaries of malignant lung and bronchus cancer (sequence numbers 0 and 1 from the cancer registry) from 1981 to 2014 were included. To be able to allow at least a 5-year follow-up for survival, the follow-up time for determining death due to cancer was extended further into the year 2019. Therefore, the timeframe for analysis includes 1981–2014 for cancer diagnosis but 1981–2019 for survival follow-up. The study flowchart specifically determining the analytical data for lung and bronchus is depicted in **Figure 1**. It is important to note that male patients diagnosed with incident cases of lung and bronchus cancer are based on the International Classification of Diseases for Oncology, third edition (ICD-O-3) primary site codes C34.0–C34.9 with the corresponding morphology codes for adenocarcinoma (8050, 8140–8147, 8211, 8250–8255, 8260, 8290–8333, 8470–8550, 8570–8574, 8576), squamous cell carcinoma (8052–8078, 8083–8183), large cell carcinoma (8012–8014, 8021, 8082), not otherwise specified (8046), and small cell carcinoma (8002, 8041–8045). Histology was further grouped into NSCLC (adenocarcinoma, squamous cell carcinoma, large cell carcinoma, not otherwise specified), and SCLC (small cell carcinoma) (26). The Surveillance, Epidemiology, and End Results (SEER) cause-specific standards based on the sequence number of lung and bronchus cancer were used to identify whether the death was due to lung and bronchus cancer (27, 28). For the purpose of this study, we will refer to lung and bronchus cancer as lung cancer.

### 2.4 Lung cancer-specific death as a primary clinical outcome

Survival from lung cancer is studied using a clinical outcome of lung-elapsed time in days from the date of lung cancer diagnosis to the date of death for patients who died due to lung cancer or the earliest of the dates of last contact or 31 December 2019 for alive patients. Other causes of death not related to lung cancer were considered censored observations. Survival time in days was converted into months and years for easy interpretation. Calculation of survival time requires complete data on dates. Therefore, to be able to keep as many patients as possible in the analytical sample, missing days or months were imputed with day 15 (middle of a month) or the month of June (middle of a year) or July 1 if both day and month were missing.

### 2.5 Longest occupation held as a primary risk factor

Participants of the National Program of Cancer Registries (NPCR) collect information about the patients (29, 30). Text indicating usual occupation (“type of job patient engaged in for the greatest number of working years”) and text indicating usual industry (“type of business or industry where patient worked in his or her usual occupation”) were collected. *Via* medical record abstraction, occupation is the type of job the individual was engaged in for the longest time prior to a cancer diagnosis and might not necessarily be the highest-paid job or the most prestigious, but the one that accounted for the greatest number of working years. The central cancer registry then applies the U.S. Census occupation and industry coding system to translate reported text fields into coded variables. This allows for a systematic standard for analyzing occupation and industry data across time and geography.

In our study (**Figure 1** and **Table 1**), the binary occupation variable (firefighter or non-firefighter) is considered the predictor variable in the regression models. Firefighters who were not identified *via* data linkage but with U.S. census-derived occupation codes in the cancer registry record as 3740, 3720, and 3750 were included as firefighters. For those who were only part of the linkage by both the Florida State FMO and LexisNexis©, they were further grouped based on whether they were career or volunteer firefighters. To be able to compare firefighters with several other occupations, non-firefighters were also regrouped as white collar (10–3540, 4700–5940), blue collar (6200–9000, 9030, 9040, 9050, 9120–9750), service (3540–3710, 3730, 3800–4650), and others/unknown (6010–6130, 9010, 9060, 9070, 9100, and unknown). Other occupations that were not grouped as broader occupational groups include but are not limited to farm workers, housewives, homemakers, students, retired, and disabled.

**Table 1:** The sources of firefighting occupation information for male lung and bronchus cancer patients (1981–2014).

Occupation		All patients	Source		Cancer registry census-derived occupation code^a^
			Linkage	FCDS	
All patients		210,541	606	209,935	
**Firefighters** *n* = 761	Career	604	449	155	3740, 3720, 3750
	Volunteer	157	157	**–**	
**Non-firefighters** *n* = 209,780	White collar	8,818	**–**	8,818	10**–**3540, 4700**–**5940
	Blue collar	10,899	**–**	10,899	6200**–**9000, 9030, 9040, 9050, 9120**–**9750
	Service	1,880	**–**	1,880	3540**–**3710, 3730, 3800**–**4650
	Others/unknown^b^	188,183	**–**	188,183	6010**–**6130, 9010, 9060, 9070, 9100, and unknown

Source: Linkage includes Florida State Fire Marshal’s Office (FMO) firefighters’ employment and certification data (1972–2012) and LexisNexis©, a national dataset of legal, government, business, and high-tech information sources used to collect missing identifiers such as firefighter date of birth and social security number. FCDS is the Florida Cancer Data System incidence cancer records (1981–2014).

a Occupation code is item #330 from the North American Association of Central Cancer Registries (NAACCR) that uses industry code from the US census (https://www.census.gov/topics/employment/industry-occupation/guidance.html).

b Others/unknown includes farm workers, housewives/homemakers, students, retired, disabled, and unknown.

### 2.6 Covariables used for adjustment

Several variables used as additional covariables included the year of cancer diagnosis, age at cancer diagnosis (years), race, ethnicity, health insurance, neighborhood-level socioeconomic status (SES), cigarette use, SEER tumor stage, histology, treatment received for surgery, radiation therapy, and chemotherapy.

### 2.7 Statistical data analysis methods

**Figure 2:**
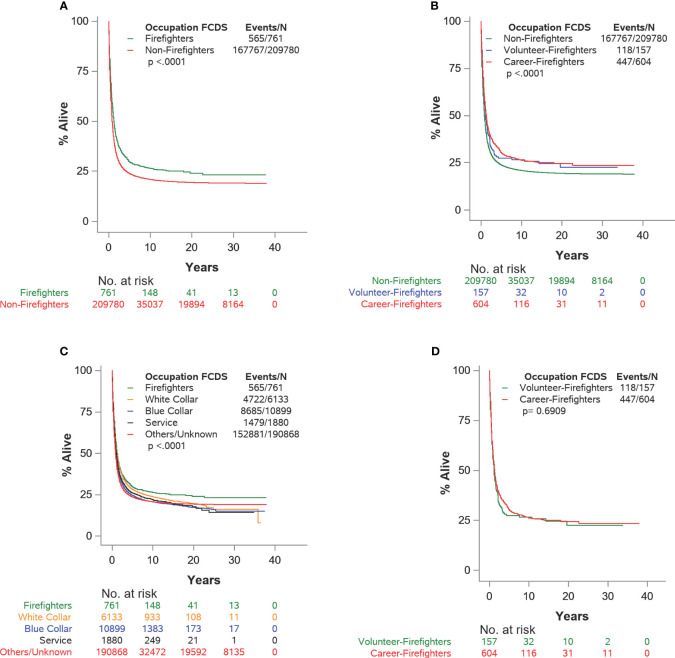
**(A–D)** Kaplan–Meier survival plots for cause-specific survival for male lung and bronchus cancer patients by detailed occupation status: Florida Cancer Data System (1981–2014).

The sources and codes for determining occupational groups were detailed (**Table 1**). The demographic and clinical characteristics of male lung cancer patients were summarized by frequencies and percentages for the entire sample and by occupation (firefighters, non-firefighters, and sub-occupational groups of non-firefighters) (**Tables 2**–**5**), and occupational group differences were compared using the chi-square test for independence. The Kaplan–Meier method for survival analysis was performed to calculate median survival time as well as the proportion of survival at 1, 3, 5, and 10 years (**Figure 2** and **Table 6**). Log-rank tests were calculated to determine any differences among occupational groups and reported with Kaplan–Meier survival curves. Univariable and multivariable Cox proportional hazard regression models were fit for lung-cancer-specific death where occupation was the main effect in the models (**Table 7**, **Supplementary Table 1**). Multivariable models included additional covariables to further adjust the differences that might happen due to year of cancer diagnosis, age at cancer diagnosis (years), race, ethnicity, health insurance, neighborhood SES, cigarette use, SEER tumor stage, histology, treatment received for surgery, radiation therapy, and chemotherapy. Unadjusted (HR) and adjusted hazard ratio (aHR) with a 95% confidence interval were calculated. Type I error was set to 5%, where a *p*-value less than 0.05 was considered statistically significant.

**Table 2:** Demographic characteristics of male lung and bronchus cancer patients by occupation: Florida Cancer Data System (1981–2014).

Characteristics	All patients		Occupation								
			Non-firefighters		Firefighters		Firefighters				
							Career			Volunteer	
	*N*	Col%	*N*	Col%	*N*	Col%	*N*	Col%		*N*	Col%
**All patients**	210,541	100.0	209,780	100.0	761	100.0	604	100.0		157	100.0
Year of cancer DX											
1981–1991	61,773	29.3	61,690	29.4	83	10.9	72	11.9		11	7.0
1992–2002	71,318	33.9	71,090	33.9	228	30.0	175	29.0		53	33.8
2003–2014	77,450	36.8	77,000	36.7	450	59.1	357	59.1		93	59.2
Age at diagnosis											
18–44	4,340	2.1	4,305	2.1	35	4.6	26	4.3		<10	–
45–54	19,575	9.3	19,473	9.3	102	13.4	77	12.7		25	15.9
55–64	50,111	23.8	49,887	23.8	224	29.4	182	30.1		42	26.8
65–74	76,965	36.6	76,703	36.6	262	34.4	201	33.3		61	38.9
75+	59,550	28.3	59,412	28.3	138	18.1	118	19.5		20	12.7
Race											
White	190,034	90.3	189,301	90.2	733	96.3	579	95.9		154	98.1
Black	18,334	8.7	18,308	8.7	26	3.4	24	4.0		<10	–
Others/unknown	2,173	1.0	2,171	1.0	<10	–	<10	–		<10	–
Ethnicity											
Non-Hispanic	193,120	91.7	192,370	91.7	750	98.6	596	98.7		154	98.1
Hispanic	14,840	7.0	14,831	7.1	<10	–	<10	–		<10	–
Unknown	2,581	1.2	2,579	1.2	<10	–	<10	–		<10	–
Insurance											
Uninsured	5,868	2.8	5,855	2.8	13	1.7	<10	–		<10	–
Insured	118,533	56.3	117,925	56.2	608	79.9	479	79.3		129	82.2
Unknown	86,140	40.9	86,000	41.0	140	18.4	116	19.2		24	15.3
SES—% poverty level											
20%–100% poverty	20,772	9.9	20,678	9.9	94	12.4	65	10.8		29	18.5
10%–<20% poverty	43,189	20.5	42,936	20.5	253	33.2	195	32.3		58	36.9
5%–<10% poverty	37,743	17.9	37,540	17.9	203	26.7	167	27.6		36	22.9
0%–<5% poverty	15,621	7.4	15,551	7.4	70	9.2	61	10.1		9	5.7
Unknown	93,216	44.3	93,075	44.4	141	18.5	116	19.2		25	15.9
Cigarette use											
Never	14,934	7.1	14,887	7.1	47	6.2	40	6.6		<10	–
History	76,586	36.4	76,289	36.4	297	39.0	232	38.4		65	41.4
Current	79,104	37.6	78,809	37.6	295	38.8	239	39.6		56	35.7
Unknown	39,917	19.0	39,795	19.0	122	16.0	93	15.4		29	18.5

All p-values for comparing firefighters vs. non-firefighters and also for comparing career firefighters vs. volunteer firefighters vs. non-firefighters are less than 0.05, i.e., statistically significant, except the following: SES % poverty level and cigarette use were not statistically significant by occupation: firefighters vs. non-firefighters (p > 0.05). Cigarette use was not statistically significant by occupation: career firefighters vs. volunteer firefighters vs. non-firefighters (p > 0.05).

DX, diagnosis; SES, socioeconomic status reported as the percent poverty level of the patients’ neighborhood at the time of cancer diagnosis; –, sample size less than 10 not reported due to confidentiality rules.

**Table 3:** Clinical characteristics of male lung and bronchus cancer patients by occupation: Florida Cancer Data System (1981–2014).

Characteristics	All patients		Occupation							
			Non-firefighters		Firefighters		Firefighters			
							Career		Volunteer	
	*N*	Col%	*N*	Col%	*N*	Col%	*N*	Col%	*N*	Col%
**All patients**	210,541	100.0	209,780	100.0	761	100.0	604	100.0	157	100.0
SEER stage										
Localized	34,078	16.2	33,959	16.2	119	15.6	99	16.4	20	12.7
Regional	49,236	23.4	49,011	23.4	225	29.6	182	30.1	43	27.4
Distant	98,403	46.7	98,045	46.7	358	47.0	274	45.4	84	53.5
Unknown	28,824	13.7	28,765	13.7	59	7.8	49	8.1	10	6.4
Surgery received										
No	163,357	77.6	162,803	77.6	554	72.8	430	71.2	124	79.0
Yes	45,996	21.8	45,793	21.8	203	26.7	171	28.3	32	20.4
Unknown	1,188	0.6	1,184	0.6	<10	–	<10	–	<10	–
Radiation received										
No	125,864	59.8	125,443	59.8	421	55.3	336	55.6	85	54.1
Yes	80,206	38.1	79,886	38.1	320	42.0	252	41.7	68	43.3
Unknown	4,471	2.1	4,451	2.1	20	2.6	16	2.6	<10	–
Chemotherapy received										
No	134,619	63.9	134,224	64.0	395	51.9	321	53.1	74	47.1
Yes	70,387	33.4	70,051	33.4	336	44.2	263	43.5	73	46.5
Unknown	5,535	2.6	5,505	2.6	30	3.9	20	3.3	10	6.4
Histology										
NSCLC	148,986	70.8	148,413	70.7	573	75.3	455	75.3	118	75.2
SCLC	29,621	14.1	29,511	14.1	110	14.5	85	14.1	25	15.9
Unspecified/unknown	31,934	15.2	31,856	15.2	78	10.2	64	10.6	14	8.9
Vital status										
Alive/dead—other causes	42,209	20.0	42,013	20.0	196	25.8	157	26.0	39	24.8
Dead—primary diagnosis	168,332	80.0	167,767	80.0	565	74.2	447	74.0	118	75.2

All p-values for comparing firefighters vs. non-firefighters and also for comparing career firefighters vs. volunteer firefighters vs. non-firefighters are less than 0.05, i.e., statistically significant, except the following: Histology was not significant by occupation: firefighters vs. non-firefighters (p > 0.05); radiation and histology were not significant by occupation: career firefighters vs. volunteer firefighters vs. non-firefighters (p > 0.05). Vital status is based on incidence cases from 1981 to 2014 with passive follow-up up to 31 December 2019, where death was due to lung and bronchus cancer.

SEER, Surveillance, Epidemiology, and End Results (SEER) Program; NSCLC, non-small cell lung cancer; SCLC, small cell lung cancer; –, sample size less than 10 not reported due to confidentiality rules.

**Table 4:** Demographics characteristics of male lung and bronchus cancer patients by detailed occupation status: Florida Cancer Data System (1981–2014).

Characteristics	All patients		Occupation											
			Non-firefighters		Firefighters		Non-firefighters							
							White collar		Blue collar		Service		Others/unknown	
	*N*	Col%	*N*	Col%	*N*	Col%	*N*	Col%	*N*	Col%	*N*	Col%	*N*	Col%
**All patients**	210,541	100.0	209,780	100.0	761	100.0	8,818	100.0	10,899	100.0	1,880	100.0	188,183	100.0
Year of cancer DX														
1981–1991	61,773	29.3	61,690	29.4	83	10.9	82	0.9	81	0.7	<10	–	61,518	32.7
1992–2002	71,318	33.9	71,090	33.9	228	30.0	2,020	22.9	2,440	22.4	329	17.5	66,301	35.2
2003–2014	77,450	36.8	77,000	36.7	450	59.1	6,716	76.2	8,378	76.9	1,542	82.0	60,364	32.1
Age at diagnosis														
18–44	4,340	2.1	4,305	2.1	35	4.6	191	2.2	286	2.6	68	3.6	3,760	2.0
45–54	19,575	9.3	19,473	9.3	102	13.4	762	8.6	1,556	14.3	279	14.8	16,876	9.0
55–64	50,111	23.8	49,887	23.8	224	29.4	2,295	26.0	3,144	28.8	566	30.1	43,882	23.3
65–74	76,965	36.6	76,703	36.6	262	34.4	2,964	33.6	3,614	33.2	603	32.1	69,522	36.9
75+	59,550	28.3	59,412	28.3	138	18.1	2,606	29.6	2,299	21.1	364	19.4	54,143	28.8
Race														
White	190,034	90.3	189,301	90.2	733	96.3	8,367	94.9	9,804	90.0	1,613	85.8	169,517	90.1
Black	18,334	8.7	18,308	8.7	26	3.4	335	3.8	979	9.0	232	12.3	16,762	8.9
Others/unknown	2,173	1.0	2,171	1.0	<10	–	116	1.3	116	1.1	35	1.9	1,904	1.0
Ethnicity														
Non-Hispanic	193,120	91.7	192,370	91.7	750	98.6	8,294	94.1	10,204	93.6	1,727	91.9	172,145	91.5
Hispanic	14,840	7.0	14,831	7.1	<10	–	486	5.5	660	6.1	147	7.8	13,538	7.2
Unknown	2,581	1.2	2,579	1.2	<10	–	38	0.4	35	0.3	<10	–	2,500	1.3
Insurance														
Uninsured	5,868	2.8	5,855	2.8	13	1.7	268	3.0	691	6.3	124	6.6	4,772	2.5
Insured	118,533	56.3	117,925	56.2	608	79.9	8,129	92.2	9,750	89.5	1,683	89.5	98,363	52.3
Unknown	86,140	40.9	86,000	41.0	140	18.4	421	4.8	458	4.2	73	3.9	85,048	45.2
SES—% poverty level														
20%–100% poverty	20,772	9.9	20,678	9.9	94	12.4	962	10.9	2,236	20.5	357	19.0	17,123	9.1
10%–<20% poverty	43,189	20.5	42,936	20.5	253	33.2	2,564	29.1	3,973	36.5	619	32.9	35,780	19.0
5%–<10% poverty	37,743	17.9	37,540	17.9	203	26.7	2,866	32.5	2,819	25.9	534	28.4	31,321	16.6
0%–<5% poverty	15,621	7.4	15,551	7.4	70	9.2	1,567	17.8	987	9.1	176	9.4	12,821	6.8
Unknown	93,216	44.3	93,075	44.4	141	18.5	859	9.7	884	8.1	194	10.3	91,138	48.4
Cigarette use														
Never	14,934	7.1	14,887	7.1	47	6.2	681	7.7	495	4.5	103	5.5	13,608	7.2
History	76,586	36.4	76,289	36.4	297	39.0	2,578	29.2	4,496	41.3	753	40.1	68,462	36.4
Current	79,104	37.6	78,809	37.6	295	38.8	4,121	46.7	4,120	37.8	698	37.1	69,870	37.1
Unknown	39,917	19.0	39,795	19.0	122	16.0	1,438	16.3	1,788	16.4	326	17.3	36,243	19.3

All p-values for comparing across occupation groups are less than 0.05, i.e., statistically significant.

DX, diagnosis; SES: socioeconomic status reported as the poverty level (percent) of the patients’ neighborhood at the time of cancer diagnosis; –, sample size less than 10 not reported due to confidentiality rules.

Data management and statistical analyses were conducted using the SAS Enterprise Guide v5.1 and SAS v9.4 for Windows (SAS Institute Inc. Cary, NC, USA). This study was approved by the Institutional Review Boards of the Florida Department of Health and the University of Miami.

## 3 Results

### 3.1 Analytical final study data consort

The study flowchart leading to the final analytical dataset is depicted in **Figure 1**. For our study, data were drawn from the aforementioned linkage, which included Florida firefighters with a cancer diagnosis (*n* = 4,943; career: 3,905; volunteer: 1,038). The merged data yielded 875,509 patients with cancer. Patients were retained if lung cancer was the first two of the reportable primary tumors and excluded if the cancer was diagnosed after they were certified as a firefighter or after the start date as a volunteer. The patients were further excluded if they were female, if they had cancer in other cancer sites, or if their tumor report source was an autopsy or only a death certificate. This resulted in *n* = 210,663 male patients with lung cancer. Further exclusions were made on patients if they had invalid follow-up time or SEER summary tumor stage *in situ* (*n* = 122). The resulting final analytic dataset contained 210,541 male patients with lung cancer stages I–IV with valid follow-up time.

### 3.2 Patients’ occupational groups

Occupation at the time of cancer diagnosis is considered a primary predictor variable that was grouped broadly first (firefighters and non-firefighters) and then with detailed occupational groups within firefighters (career, volunteer) and non-firefighters (white collar, blue collar, services, others/unknown) (**Table 1**). Out of the 210,541 male lung cancer patients, there were 99.6% non-firefighters and 0.4% (*n* = 761) firefighters [career: 604 (79.4%); volunteer: 157 (20.6%)]. Categorization of the longest-held job was not possible for nearly 90% of patients due to missing information or because they fell into the following categories: farm worker, housewife, homemaker, student, retired, and disabled.

### 3.3 Demographic and clinical characteristics of the patients by occupational groups

The demographic (**Table 2**) and clinical (**Table 3**) characteristics of the patients by firefighters and non-firefighters, along with broad occupational groups, were summarized by descriptive statistics.

Overall, the majority of the patients who were diagnosed during 2003–2014 (36.8%) were between 65 and 74 years old when diagnosed (36.6%); were white (90.3%) and non-Hispanic (91.7%); were insured (56.3%); were living in a neighborhood with a poverty level between 10% and 20% (20.5%); were ever smokers (74%; 37.6% current and 36.4% past smoker); had distant SEER stage (46.7%); had not received surgery (77.6%), radiation (59.8%), or chemotherapy (63.9%); had non-small cell lung cancer (NSCLC, 70.8%); and died due to lung cancer (80.0%).

Compared with non-firefighters (non-FF), a higher proportion of firefighters (FF) who were diagnosed between 2003 and 2014 (FF: 59.1% *vs*. non-FF: 36.7%) were diagnosed before the age of 65 years (47.4% *vs*. 35.2%), were insured (79.9% *vs*. 56.2%), were living in a neighborhood with poverty level ≥20% (12.4% *vs*. 9.9%), and were ever smokers, i.e., current and history (77.8% *vs*. 74%; current: 38.8% *vs*. 37.6% and history: 39% *vs*. 36.4%). However, there were fewer black (3.4% *vs*. 8.7%) and Hispanic (1.2% vs. 7.1%) patients among firefighters than among non-firefighters.

Compared with non-firefighters, a higher proportion of firefighters were diagnosed with regional (FF: 29.6% *vs*. non-FF: 23.4%) and distant (47% *vs*. 46.7%) tumor stage; received surgery (26.7% *vs*. 21.8%), radiation therapy (42% *vs*. 38.1%), or chemotherapy (44.2% *vs*. 33.4%); and diagnosed with NSCLC (75.3% *vs*. 70.7%). However, there were fewer lung cancer-related deaths (74.2% *vs*. 80%) among firefighters than among non-firefighters during the study period.

To be able to compare firefighters with different occupational groups, non-firefighters were further grouped into white collar, blue collar, service, and others/unknown. The demographic (**Table 4**) and clinical (**Table 5**) characteristics of the patients belonging to the firefighters group and the different occupational sectors were summarized by descriptive statistics.

**Table 5:** Clinical characteristics of male lung and bronchus cancer patients by detailed occupation status: Florida Cancer Data System (1981–2014).

Characteristics	All patients		Occupation											
			Non-firefighters		Firefighters		Non-firefighters							
							White collar		Blue collar		Service		Others/unknown	
	*N*	Col%	*N*	Col%	*N*	Col%	*N*	Col%	*N*	Col%	*N*	Col%	*N*	Col%
**All patients**	210,541	100.0	209,780	100.0	761	100.0	8,818	100.0	10,899	100.0	1,880	100.0	188,183	100.0
SEER stage														
Localized	34,078	16.2	33,959	16.2	119	15.6	1,531	17.4	1,567	14.4	284	15.1	30,577	16.2
Regional	49,236	23.4	49,011	23.4	225	29.6	2,277	25.8	2,841	26.1	493	26.2	43,400	23.1
Distant	98,403	46.7	98,045	46.7	358	47.0	4,506	51.1	5,864	53.8	1,012	53.8	86,663	46.1
Unknown	28,824	13.7	28,765	13.7	59	7.8	504	5.7	627	5.8	91	4.8	27,543	14.6
Surgery received														
No	163,357	77.6	162,803	77.6	554	72.8	6,395	72.5	8,557	78.5	1,451	77.2	146,400	77.8
Yes	45,996	21.8	45,793	21.8	203	26.7	2,372	26.9	2,276	20.9	416	22.1	40,729	21.6
Unknown	1,188	0.6	1,184	0.6	<10	–	51	0.6	66	0.6	13	0.7	1,054	0.6
Radiation received														
No	125,864	59.8	125,443	59.8	421	55.3	4,906	55.6	5,758	52.8	961	51.1	113,818	60.5
Yes	80,206	38.1	79,886	38.1	320	42.0	3,679	41.7	4,821	44.2	854	45.4	70,532	37.5
Unknown	4,471	2.1	4,451	2.1	20	2.6	233	2.6	320	2.9	65	3.5	3,833	2.0
Chemotherapy received														
No	134,619	63.9	134,224	64.0	395	51.9	3,965	45.0	4,871	44.7	763	40.6	124,625	66.2
Yes	70,387	33.4	70,051	33.4	336	44.2	4,539	51.5	5,606	51.4	1,045	55.6	58,861	31.3
Unknown	5,535	2.6	5,505	2.6	30	3.9	314	3.6	422	3.9	72	3.8	4,697	2.5
Histology														
NSCLC	148,986	70.8	148,413	70.7	573	75.3	7,072	80.2	8,385	76.9	1,475	78.5	131,481	69.9
SCLC	29,621	14.1	29,511	14.1	110	14.5	1,014	11.5	1,574	14.4	268	14.3	26,655	14.2
Unspecified/unknown	31,934	15.2	31,856	15.2	78	10.2	732	8.3	940	8.6	137	7.3	30,047	16.0
Vital status														
Alive/dead—other causes	42,209	20.0	42,013	20.0	196	25.8	2,017	22.9	2,214	20.3	401	21.3	37,381	19.9
Dead—primary diagnosis	168,332	80.0	167,767	80.0	565	74.2	6,801	77.1	8,685	79.7	1,479	78.7	150,802	80.1

All p-values for comparing across occupation groups are less than 0.05, i.e., statistically significant. Vital status is based on incidence cases from 1981 to 2014 with passive follow-up up to 31 December 2019, where death was due to lung and bronchus cancer.

SEER, Surveillance, Epidemiology, and End Results (SEER) Program; NSCLC, non-small cell lung cancer; SCLC, small cell lung cancer; –, sample size less than 10 not reported due to confidentiality rules.

### 3.4 Cause-specific overall survival estimates

Surviving proportions at 1, 3, 5, and 10 years and the median survival of cause-specific overall survival by detailed occupational groups are summarized in **Table 6**. For all patients, the 1-, 3-, 5-, and 10-year survival rates were 44.6%, 27.4%, 23.8%, and 20.9%, respectively. Firefighters have a higher proportion of survival than non-firefighters at 1, 3, 5, and 10 years (FF: 54.4%, 34.8%, 29.7%, 26.3% *vs*. 44.6%, 27.4%, 23.8%, 20.9%, respectively). Career firefighters have a higher proportion of survival than non-firefighters at 1, 3, 5, and 10 years (career: 55%, 35.8%, 30.3% *vs*. volunteer: 52.9%, 31.2%, 27.4%, respectively) but slightly lower at 10 years (26.2% *vs*. 26.6%).

**Table 6:** Survival proportions and median cause-specific survival time for male lung and bronchus cancer patients by detailed occupation status: Florida Cancer Data System (1981–2014).

Occupation	*N*	Survival proportions (%) with 95% confidence interval				Median survival (years)
		1 year	3 years	5 years	10 years	
**All patients**	210,541	44.6 (44.4–44.8)	27.4 (27.3–27.6)	23.8 (23.6–24.0)	20.9 (20.7–21.0)	0.1 (–)
**All firefighters**	761	54.5 (50.9–58.0)	34.8 (31.5–38.2)	29.7 (26.5–33.0)	26.3 (23.2–29.5)	0.1 (0.1–0.1)
Career	604	55.0 (50.9–58.8)	35.8 (32.0–39.6)	30.3 (26.7–34.0)	26.2 (22.7–29.8)	0.1 (0.1–0.1)
Volunteer	157	52.9 (44.8–60.3)	31.2 (24.1–38.5)	27.4 (20.7–34.5)	26.6 (20.0–33.7)	0.1 (0.1–0.1)
**All non-firefighters**	209,780	44.6 (44.4–44.8)	27.4 (27.2–27.6)	23.8 (23.6–24.0)	20.9 (20.7–21.0)	0.1 (0.1–0.1)
White collar	8,818	52.7 (51.6–53.7)	33.3 (32.3–34.3)	28.0 (27.1–28.9)	23.5 (22.6–24.4)	0.1 (0.1–0.1)
Blue collar	10,899	47.9 (47.0–48.8)	28.9 (28.1–29.8)	24.6 (23.8–25.4)	20.6 (19.9–21.4)	0.1 (0.1–0.1)
Service	1,880	50.1 (47.8–52.3)	30.8 (28.7–32.9)	26.0 (24.1–28.0)	21.9 (20.0–23.8)	0.1 (0.1–0.1)
Others/unknown	188,183	44.0 (43.7–44.2)	27.0 (26.8–27.2)	23.5 (23.3–23.7)	20.7 (20.5–20.9)	0.1 (NE–NE)

Survival estimates are based on incidence cases from 1981 to 2014 with passive follow-up up to 31 December 2019 for determining vital status.

NE, not estimable.

The Kaplan–Meier survival curves are depicted in **Figure 2** based on the different levels of occupational groups. There were statistically significant differences between firefighters and non-firefighters (log-rank *p*-value < 0.001; **Figure 2A**), among non-firefighters and career firefighters and volunteer firefighters (*p*-value < 0.001; **Figure 2B**), and among firefighters and the blue collar, white collar, service, and others/unknown occupational groups (*p*-value < 0.001; **Figure 2C**). However, there were no significant differences between career firefighters and volunteer firefighters (*p*-value = 0.6909; **Figure 2D**).

### 3.5 Cause-specific overall survival regression models

The univariable and multivariable Cox proportional hazard regression models for cause-specific overall survival for male lung cancer patients with occupational groups as the main effect are summarized in **Table 7** and **Supplementary Table 1**.

In the univariable models, compared with non-firefighters, firefighters have significantly higher cause-specific survival (HR = 0.82; 95% CI: 0.75–0.89); *p* < 0.001). Both career firefighters (0.81; 0.74–0.89; *p* < 0.001) and volunteer firefighters (0.84; 0.70–1.01; *p* = 0.063) have higher cause-specific survival, but only career firefighters were significantly different from non-firefighters. There were no significant differences in cause-specific survival between career and volunteer firefighters (0.96; 0.79–1.18; p=0.718). Compared with firefighters, the blue collar (1.17; 1.08–1.28; *p* < 0.001), service (1.13; 1.02–1.24; *p* = 0.016), and others/unknown (1.23; 1.14-1.34; *p* < 0.001) occupational groups had significantly lower cause-specific survival, but the difference for white collar workers failed to reach statistical significance (1.06; 0.98–1.16; *p* = 0.166).

Two multivariable models (**Table 7**, **Supplementary Table 1**) with two different occupational groups as the main effect variable were fit to account for the differences that might occur due to year of cancer diagnosis, age at cancer diagnosis (years), race, ethnicity, health insurance, neighborhood SES, cigarette use, SEER tumor stage, histology, treatment received as surgery, radiation therapy, and chemotherapy.

**Table 7:** Cox proportional hazard regression models for cause-specific survival for male lung and bronchus cancer patients by detailed occupation status: Florida Cancer Data System (1981–2014).

Occupation	Univariate models		Multivariable models			
			Model 1		Model 2	
	HR (95% CI)	*p*-value	aHR (95% CI)	*p*-value	aHR (95% CI)	*p*-value
Non-firefighters	1.00 (reference)		1.00 (reference)		–	
Firefighters	0.82 (0.75, 0.89)	<0.001	0.84 (0.77, 0.91)	<0.001	–	
Non-firefighters	1.00 (reference)		1.00 (reference)		–	
Volunteer firefighters	0.84 (0.70, 1.01)	0.063	0.76 (0.63, 0.91)	0.003	–	
Career firefighters	0.81 (0.74, 0.89)	<0.001	0.86 (0.79, 0.95)	0.002	–	
Volunteer firefighters	1.00 (reference)		1.00 (reference)		–	
Career firefighters	0.96 (0.79, 1.18)	0.718	1.14 (0.93, 1.39)	0.213	–	
Firefighters	1.00 (reference)		1.00 (reference)		–	
Non-firefighters	1.22 (1.13, 1.33)	<0.001	1.20 (1.10, 1.30)	<0.001	–	
Firefighters	1.00 (reference)		–		1.00 (reference)	
White collar	1.06 (0.98, 1.16)	0.166	–		1.11 (1.02, 1.21)	0.016
Blue collar	1.17 (1.08, 1.28)	<0.001	–		1.15 (1.05, 1.25)	0.002
Service	1.13 (1.02, 1.24)	0.016	–		1.13 (1.03, 1.25)	0.012
Others/unknown	1.23 (1.14, 1.34)	<0.001	–		1.21 (1.12, 1.32)	<0.001

The multivariable models are additionally adjusted with variables such as year of cancer diagnosis, age at diagnosis, race, ethnicity, insurance, SES, cigarette use, SEER stage, histology, surgery as a treatment received, radiation therapy received, and chemotherapy received. Estimates for the variables used for adjustment were provided in **Supplementary Table 1**.

–, not applicable; HR, hazard ratio; aHR, adjusted hazard ratio; 95% CI, 95% confidence interval.

In multivariable model 1 (**Table 7**, **Supplementary Table 1**), the main effect was the three-level occupational variable (non-firefighters, career firefighters, volunteer firefighters). Compared with non-firefighters, firefighters have significantly higher cause-specific survival (adjusted HR = 0.84; 95% CI: 0.77–0.91; *p* < 0.001). Both career firefighters (0.86; 0.79–0.95; *p* = 0.002) and volunteer firefighters (0.76; 0.63–0.91; *p* = 0.003) have significantly higher cause-specific survival than non-firefighters. Career firefighters have lower cause-specific survival than volunteer firefighters (1.14; 0.93–1.39; *p* = 0.213), but the result was not statistically significant.

In multivariable model 2 (**Table 4**, **Supplementary Table 1**), the main effect was the five-level occupational variable (firefighters, white collar, blue collar, service, others/unknown). Compared with firefighters, the white collar (adjusted HR = 1.11; 95% CI: 1.02–1.21; *p* = 0.016), blue collar (1.15; 1.05–1.25; *p* = 0.002), service (1.13; 1.03–1.25; *p* = 0.012), and others/unknown (1.21; 1.12–1.32; *p* < 0.001) occupational groups have significantly lower cause-specific survival.

## 4 Discussion

Our results suggest that Florida male firefighters had significantly higher cause-specific survival than non-firefighters. When compared with different non-firefighting sub-occupational groups, firefighters had significantly higher cause-specific survival. Among firefighters, although career firefighters had lower cause-specific survival than volunteer firefighters, this difference was not statistically significant.

Smoking is the leading cause of lung cancer and makes cancer treatments work less effectively (31). In the study, among patients with known smoking status, 74% were ever smokers (37.6% current and 36.4% past smokers). Although never and current smokers were slightly higher among firefighters, past smokers accounted for 39% of firefighters and 36.4% of non-firefighters. In Florida, a restrictive tobacco use hiring policy for firefighters took effect in 1989 and prohibited the hiring of firefighters that used tobacco products. Since 1989, all newly certified firefighters have been required to submit a sworn affidavit attesting that they have been a non-user of tobacco or tobacco products for at least the 12-month period immediately preceding the application (32, 33). The 1989 Florida law targeting firefighters is not an outright ban on the use of tobacco products once hired but rather focuses on tobacco use practices in the 12 months prior to the signing of the affidavit. There were no restrictions placed on currently serving firefighters, and once hired, firefighters that signed the affidavit were not bound to remain smoke-free. One possible explanation for the better survival among firefighters for a leading tobacco-associated cancer may be due to the implementation of a restrictive State of Florida tobacco use hiring policy in 1989, which led to a workplace climate that discouraged smoking.

Our analysis of data from 2013, 2015, 2016, and 2017 of the Florida Behavioral Risk Factor Surveillance System (BRFSS) includes the most recent years that featured the collection of industry and occupation data. The pooled BRFSS cigarette smoking rate for all Florida workers except firefighters is 16.7% (95% CI: 15.8–17.5). The estimated smoking rate in Florida firefighters is markedly lower (1.8%; 95% CI: 0.0–3.5) than all the other workers and for blue collar (25.4%; 23.1–27.8), service (19.6%; 17.7–21.6), and white collar (12.2%; 11.3–13.1). As reported in 2010–2011, the smoking prevalence rate had dropped to 9.8% in a non-random sample of 20 US fire departments (34). From 1992 to 2019, data from the Tobacco Use Supplement to the Current Population Survey (TUS‐CPS) found that the smoking prevalence among firefighters declined (annual percentage change: −5.0%; 95% CI: −7.7% to −2.3%) (35). For broader US worker groups for the years 2014–2016, compared with nationally representative estimates of smoking rates, Florida firefighters had rates of smoking that were lower than for all worker occupational groups including workers employed in the life, physical, and social sciences (5.6%; 95% CI: 3.5–7.7) (36).

Improved survival for firefighters might be due to a healthy worker effect or possibly superior treatment compliance (37). Given that many firefighters are also cross-trained as EMTs/paramedics, they may have a level of medical knowledge that may favorably impact treatment engagement and better navigation to complex cancer care. In our study, we found that compared with non-firefighters, a higher proportion of firefighters received surgery (26.7% *vs*. 21.8%), radiation therapy (42% *vs*. 38.1%), and chemotherapy (44.2% *vs*. 33.4%).

### 4.1 Limitations and strengths

This is an observational retrospective cohort study from a statewide cancer registry that is enhanced with firefighter employee and certification data together with a national dataset of legal, government, business, and high-tech information sources and the state office of vital statistics and national death index.

This is the first population-based epidemiological study of cause-specific overall survival of those with a lung and bronchus cancer diagnosis comparing firefighters with non-firefighters and subgroups of non-firefighters in Florida. Our study is unique since we also included volunteer firefighters in our sample to be able to compare the differences between career and volunteer firefighters’ lung cancer cause-specific survival.

This study is not without limitations. It is based on cancer records from a population-based cancer registry with passive follow-up for survival. Although the sample size was increased by including information from cancer registry occupation records, some of the other/unknown occupation categories might include retired firefighters who did not report their firefighting status. Information on occupation in cancer registries is also incomplete and inaccurate, which may have limited our ability to make comparisons with other broad worker groups (38).

Information on occupational carcinogenic exposure or the number of years in a firefighting career was not available when the analysis focused only on firefighters. The completeness of smoking status suffered from missing values. When the smoking status was known, the length of smoking quit time for past smokers was not known. Therefore, this limits our understanding of smoking cessation in lung cancer survival among firefighters compared with all the other occupational groups.

The results from this unique enhanced dataset should be interpreted cautiously due to limitations of individual exposure data both for firefighters and non-firefighters. Confounding factors such as time to any first cancer treatment and its duration and health behaviors that may influence cancer survival were not adjusted in all analyses, mostly due to limitation or non-existence of the related variables.

Female firefighters were not included in the analysis due to their small numbers. Therefore, further epidemiological research for lung cancer survival among female firefighters is warranted.

### 4.2 Conclusion

Firefighting is a hazardous occupation and firefighters face unique occupational exposures. Lung cancer survival was significantly higher among firefighters compared with non-firefighters. Compared with non-firefighters, firefighters had a statistically significant 16% (career 14%, volunteer 24%) lower risk of lung cancer cause-specific mortality. However, there were no significant survival differences between career and volunteer firefighters, even if career firefighters had a 14% higher risk of lung cancer cause-specific mortality than volunteer firefighters. Improved survival for firefighters might be due to a healthy worker effect to start with before cancer diagnosis or lower smoking rates among them and possibly superior treatment compliance and engagement with better navigation to complex cancer care. Given that many firefighters are also cross-trained as EMTs/paramedics, they may have a level of medical knowledge that may favorably impact treatment engagement and better navigation to complex cancer care. Additional epidemiological studies are needed to determine and link occupational and environmental exposure data to cancer cohort studies including female firefighters.

## Data availability statement

The datasets presented in this article are not readily available because the datasets collected and analyzed for this study are not publicly available due to strict confidentiality agreements between the University of Miami and the Florida Department of Health, Florida Cancer Data System, Florida Fire Marshalls Office. This study was approved by the Institutional Review Boards of the Florida Department of Health and the University of Miami. A waiver of informed consent was granted given that cancer data is a reportable event for the purposes of cancer surveillance. Requests to access the datasets should be directed to the Florida Department of Health, health@flhealth.gov.

## Ethics statement

This study was approved by the Institutional Review Boards of the Florida Department of Health and the University of Miami. A waiver of informed consent was granted given that cancer data is a reportable event for the purposes of cancer surveillance. Written informed consent for participation was not required for this study in accordance with national legislation and institutional requirements.

## Author contributions

Study concept and design: TK-S, PP, and DL. Acquisition, analysis, and interpretation of data: TK-S, PP, WZ, and DH. Drafting of the manuscript: TK-S. Critical revision of the manuscript for important intellectual content: all authors. Statistical analysis: TK-S, WZ, DH, and PP. Obtained funding: EK. Administrative, technical, or material support: TK-S, DL, AC-M, and EK. Study supervision: TK-S, PP, and DL. All authors participated in the revision of the manuscript for important intellectual content and consented to the publication of this submitted manuscript. All authors agree to be accountable for all aspects of the work in ensuring that questions related to the accuracy or integrity of any part of the work are appropriately investigated and resolved.

## Supplementary material

Supplementary Table 1 Cox Proportional Hazard Regression Models for Cause-Specific Survival for Male Lung and Bronchus Cancer Patients by Detailed Occupation Status: Florida Cancer Data System (1981-2014).
