# Supplementary material for: Lung cancer survival among Florida male firefighters
Source: Front Oncol. 2023 Aug 18;13:1155650. doi: 10.3389/fonc.2023.1155650 (PMC10473410; doi:10.3389/fonc.2023.1155650)
Supplement: Supplementary Table 1 — Cox Proportional Hazard Regression Models for Cause-Specific Survival for Male Lung and Bronchus Cancer Patients by Detailed Occupation Status: Florida Cancer Data System (1981-2014). [file Table_1.docx]

**Supplementary Table 1.** Cox Proportional Hazard Regression Models for Cause-Specific Survival for Male Lung and Bronchus Cancer Patients by Detailed Occupation Status: Florida Cancer Data System (1981-2014)

|  |  | **Univariate Models** | | | **Multivariable Model 1** | | **Multivariable Model 2** | |  |
| --- | --- | --- | --- | --- | --- | --- | --- | --- | --- |
| **Variables** | **Category** | **HR (95%CI)*** | **p-value** | **aHR (95%CI)*** | | **p-value** | **aHR (95%CI)*** | **p-value** |  |
| **Occupation** | Non-Firefighters | 1.00 (Reference) |  | NA | |  | 1.00 (Reference) |  |  |
|  | Volunteer-Firefighters | 0.84 (0.70, 1.01) | 0.063 |  | |  | 0.76 (0.63, 0.91) | 0.003 |  |
|  | Career-Firefighters | 0.81 (0.74, 0.89) | <0.001 |  | |  | 0.86 (0.79, 0.95) | 0.002 |  |
|  |  |  |  |  | |  |  |  |  |
|  | Volunteer-Firefighters | 1.00 (Reference) |  | NA | |  | 1.00 (Reference) |  |  |
|  | Career-Firefighters | 0.96 (0.79, 1.18) | 0.718 |  | |  | 1.14 (0.93, 1.39) | 0.213 |  |
|  |  |  |  |  | |  |  |  |  |
|  | Non-Firefighters | 1.00 (Reference) |  | NA | |  | 1.00 (Reference) |  |  |
|  | Firefighters | 0.82 (0.75, 0.89) | <0.001 |  | |  | 0.84 (0.77, 0.91) | <0.001 |  |
|  |  |  |  |  | |  |  |  |  |
| **Occupation** | Firefighters | 1.00 (Reference) |  | 1.00 (Reference) | |  | NA |  |  |
|  | White Collar | 1.06 (0.98, 1.16) | 0.166 | 1.11 (1.02, 1.21) | | 0.016 |  |  |  |
|  | Blue Collar | 1.17 (1.08, 1.28) | <0.001 | 1.15 (1.05, 1.25) | | 0.002 |  |  |  |
|  | Service | 1.13 (1.02, 1.24) | 0.016 | 1.13 (1.03, 1.25) | | 0.012 |  |  |  |
|  | Others/Unknown | 1.23 (1.14, 1.34) | <0.001 | 1.21 (1.12, 1.32) | | <0.001 |  |  |  |
| **Occupation** | Firefighters | 1.00 (Reference) |  | NA | |  | 1.00 (Reference) |  |  |
|  | Non-Firefighters | 1.22 (1.13, 1.33) | <0.001 |  | |  | 1.20 (1.10, 1.30) | <0.001 |  |
| **Year of diagnosis** | 1981-1991 | 1.00 (Reference) |  | 1.00 (Reference) | |  | 1.00 (Reference) |  |  |
|  | 1992-2002 | 0.99 (0.98, 1.01) | 0.318 | 1.03 (1.01, 1.05) | | 0.001 | 1.03 (1.01, 1.05) | 0.002 |  |
|  | 2003-2014 | 0.94 (0.93, 0.95) | <0.001 | 0.92 (0.90, 0.94) | | <0.001 | 0.91 (0.89, 0.93) | <0.001 |  |
| **Age at diagnosis** | 18-44 | 1.00 (Reference) |  | 1.00 (Reference) | |  | 1.00 (Reference) |  |  |
|  | 45-54 | 1.12 (1.08, 1.16) | <0.001 | 1.12 (1.08, 1.16) | | <0.001 | 1.12 (1.08, 1.16) | <0.001 |  |
|  | 55-64 | 1.09 (1.05, 1.13) | <0.001 | 1.15 (1.11, 1.19) | | <0.001 | 1.15 (1.11, 1.19) | <0.001 |  |
|  | 65-74 | 1.05 (1.01, 1.09) | 0.006 | 1.19 (1.15, 1.23) | | <0.001 | 1.19 (1.15, 1.23) | <0.001 |  |
|  | 75+ | 1.13 (1.10, 1.17) | <0.001 | 1.28 (1.24, 1.33) | | <0.001 | 1.29 (1.24, 1.33) | <0.001 |  |
| **Race** | NHW | 1.00 (Reference) |  | 1.00 (Reference) | |  | 1.00 (Reference) |  |  |
|  | Black | 1.08 (1.06, 1.10) | <0.001 | 0.97 (0.95, 0.98) | | <0.001 | 0.97 (0.95, 0.99) | <0.001 |  |
|  | Other/Unknown | 0.83 (0.79, 0.87) | <0.001 | 0.80 (0.76, 0.84) | | <0.001 | 0.80 (0.76, 0.85) | <0.001 |  |
| **Ethnicity** | Non-Hispanic | 1.00 (Reference) |  | 1.00 (Reference) | |  | 1.00 (Reference) |  |  |
|  | Hispanic | 0.92 (0.90, 0.93) | <0.001 | 0.92 (0.90, 0.94) | | <0.001 | 0.92 (0.90, 0.94) | <0.001 |  |
|  | Unknown | 1.11 (1.06, 1.16) | <0.001 | 1.11 (1.06, 1.16) | | <0.001 | 1.11 (1.06, 1.16) | <0.001 |  |
| **Insurance** | Uninsured | 1.00 (Reference) |  | 1.00 (Reference) | |  | 1.00 (Reference) |  |  |
|  | Insured | 0.82 (0.80, 0.85) | <0.001 | 0.97 (0.94, 1.00) | | 0.076 | 0.97 (0.94, 1.00) | 0.072 |  |
|  | Unknown | 0.88 (0.85, 0.91) | <0.001 | 1.08 (1.04, 1.11) | | <0.001 | 1.08 (1.04, 1.12) | <0.001 |  |
| **SES** | 20% - 100% poverty | 1.00 (Reference) |  | 1.00 (Reference) | |  | 1.00 (Reference) |  |  |
|  | 10% - <20% poverty | 0.97 (0.96, 0.99) | 0.004 | 0.99 (0.97, 1.01) | | 0.250 | 0.99 (0.97, 1.01) | 0.224 |  |
|  | 5% - <10% poverty | 0.93 (0.91, 0.94) | <0.001 | 0.96 (0.94, 0.98) | | <0.001 | 0.96 (0.94, 0.98) | <0.001 |  |
|  | 0% - <5% poverty | 0.87 (0.85, 0.89) | <0.001 | 0.92 (0.90, 0.94) | | <0.001 | 0.91 (0.89, 0.94) | <0.001 |  |
|  | Unknown | 0.95 (0.93, 0.97) | <0.001 | 0.88 (0.86, 0.90) | | <0.001 | 0.89 (0.87, 0.91) | <0.001 |  |
| **Cigarette use** | Never | 1.00 (Reference) |  | 1.00 (Reference) | |  | 1.00 (Reference) |  |  |
|  | History | 1.22 (1.19, 1.24) | <0.001 | 1.18 (1.16, 1.21) | | <0.001 | 1.18 (1.16, 1.21) | <0.001 |  |
|  | Current | 1.04 (1.02, 1.06) | <0.001 | 1.07 (1.05, 1.09) | | <0.001 | 1.07 (1.05, 1.09) | <0.001 |  |
|  | Unknown | 1.10 (1.08, 1.12) | <0.001 | 1.10 (1.07, 1.12) | | <0.001 | 1.10 (1.08, 1.12) | <0.001 |  |
| **SEER stage** | Localized | 1.00 (Reference) |  | 1.00 (Reference) | |  | 1.00 (Reference) |  |  |
|  | Regional | 1.88 (1.85, 1.91) | <0.001 | 1.65 (1.62, 1.68) | | <0.001 | 1.65 (1.62, 1.68) | <0.001 |  |
|  | Distant | 3.73 (3.67, 3.79) | <0.001 | 2.85 (2.80, 2.90) | | <0.001 | 2.84 (2.79, 2.89) | <0.001 |  |
|  | Unknown | 1.82 (1.79, 1.86) | <0.001 | 1.32 (1.29, 1.35) | | <0.001 | 1.32 (1.30, 1.35) | <0.001 |  |
| **Surgery** | No | 1.00 (Reference) |  | 1.00 (Reference) | |  | 1.00 (Reference) |  |  |
|  | Yes | 0.38 (0.38, 0.39) | <0.001 | 0.51 (0.50, 0.52) | | <0.001 | 0.51 (0.50, 0.52) | <0.001 |  |
|  | Unknown | 0.68 (0.63, 0.72) | <0.001 | 0.94 (0.88, 1.01) | | 0.084 | 0.95 (0.88, 1.01) | 0.097 |  |
| **Radiation therapy** | No | 1.00 (Reference) |  | 1.00 (Reference) | |  | 1.00 (Reference) |  |  |
|  | Yes | 1.27 (1.25, 1.28) | <0.001 | 0.99 (0.98, 1.00) | | 0.089 | 0.99 (0.98, 1.00) | 0.039 |  |
|  | Unknown | 0.93 (0.89, 0.96) | <0.001 | 0.94 (0.91, 0.98) | | 0.002 | 0.94 (0.91, 0.98) | 0.002 |  |
| **Chemotherapy** | No | 1.00 (Reference) |  | 1.00 (Reference) | |  | 1.00 (Reference) |  |  |
|  | Yes | 1.21 (1.20, 1.22) | <0.001 | 0.86 (0.85, 0.87) | | <0.001 | 0.86 (0.85, 0.87) | <0.001 |  |
|  | Unknown | 1.08 (1.05, 1.11) | <0.001 | 0.95 (0.91, 0.98) | | <0.001 | 0.95 (0.91, 0.98) | <0.001 |  |
| **Histology** | NSCLC | 1.00 (Reference) |  | 1.00 (Reference) | |  | 1.00 (Reference) |  |  |
|  | SCLC | 1.43 (1.41, 1.45) | <0.001 | 1.09 (1.08, 1.11) | | <0.001 | 1.09 (1.08, 1.11) | <0.001 |  |
|  | Unspecified/Unknown | 1.06 (1.04, 1.07) | <0.001 | 0.85 (0.84, 0.86) | | <0.001 | 0.85 (0.84, 0.87) | <0.001 |  |
| * HR: Hazard Ratio; aHR: Adjusted Hazard Ratio; 95%CI: 95% confidence interval. | | | | | | | | | |
